# CPCM/OSS Backfill Materials: Enhanced Thermal Properties and Heat Transfer Performance for Ground Heat Exchangers in Ground Source Heat Pump Systems

**DOI:** 10.3390/molecules31111892

**Published:** 2026-06-01

**Authors:** Dongyi Zhou, Fanchen Zhou, Jiawei Yuan, Yicai Liu

**Affiliations:** 1 School of Energy and Mechanical Engineering, Hunan University of Humanities, Science and Technology, Loudi 417000, China; jufang802884@163.com (F.Z.); y951723@163.com (J.Y.); 2 School of Energy Science and Engineering, Central South University, Changsha 410083, China

**Keywords:** phase change material (PCM), thermal energy storage (TES), composite backfill, ground source heat pump (GSHP), numerical simulation

## Abstract

This study focuses on optimizing backfill materials to enhance the heat transfer performance of ground heat exchangers (GHEs) in ground source heat pump (GSHP) systems. A series of composite phase change material/original sand soil (CPCM/OSS) backfill materials was prepared using capric acid–myristic acid/expanded graphite (CA-MA/EG) at mass ratios of 5%, 10%, 15%, and 20%. Thermal conductivity testing, differential scanning calorimetry (DSC) and thermogravimetric analysis (TGA), laboratory heat transfer tests, and 3D numerical simulations under typical intermittent summer conditions were systematically conducted. The results show that thermal conductivity, specific heat capacity, and thermal storage coefficient all increase with rising moisture content and CPCM dosage. The newly developed CPCM/OSS backfill material significantly improves the heat transfer performance of GHEs. Comprehensive thermophysical characterization indicates that the 10 wt% CPCM sample is the optimal formulation. Laboratory tests demonstrate that, relative to pure OSS backfill, the 10 wt% CPCM-doped CPCM/OSS raises the average soil temperature by approximately 2.5–2.8 °C. Numerical simulations over three consecutive days show that, relative to pure OSS backfill, the 10 wt% CPCM-doped composite enhances the heat exchange capacity per linear meter of the GHEs by 8.8%. The newly developed CPCM/OSS backfill material significantly improves the heat transfer performance of GHEs. It provides a feasible material solution and technical reference for GSHP system design.

## 1. Introduction

Energy conservation and emission reduction in the construction sector are core drivers of energy structure transition. By effectively reducing life-cycle energy use, they stimulate innovation in energy-saving technologies and promote the development of green building materials along industrial chains [1,2,3]. Ultimately, these efforts underpin the creation of a sustainable energy system. Ground Source Heat Pump (GSHP) systems are widely regarded as an ideal technical solution for building heating and cooling. Distinguished by their high energy utilization efficiency and near-zero carbon emissions during operation, they represent a pivotal advancement in sustainable building technologies [4,5,6,7]. As the core component of GSHP systems, Ground Heat Exchangers (GHEs) directly determine the operational efficiency and energy consumption level of the entire system through their heat transfer performance [8,9,10]. Meanwhile, the backfill material for GHEs acts as the heat transfer medium connecting the exchanger tube wall and the surrounding soil, with its thermophysical properties exerting a decisive effect on the heat transfer efficiency between the exchanger and the stratum [11,12]. Optimizing the heat transfer performance of GHEs not only effectively curtails the energy consumption of GSHP units but also boosts the system coefficient of performance, thereby propelling overall building energy efficiency.

Currently, research on backfill materials for vertical GHEs remains relatively scarce, creating a significant bottleneck for the advancement of GSHP technology. Therefore, developing backfill materials that balance geological adaptability, high thermal conductivity, cost-effectiveness, and durability is crucial for the widespread adoption of GSHP systems [13,14]. Traditional GHEs backfill materials mainly include bentonite, cement mortar, or original sand soil, which generally suffer from inherent defects of low thermal conductivity and poor heat storage capacity. Over time, these limitations trigger a “heat/cold accumulation” effect around the exchanger. This imbalance in stratum temperature not only degrades heat exchange efficiency but also shortens the system’s lifespan [15,16]. To overcome this, researchers have turned to Phase Change Materials (PCMs). By absorbing or releasing latent heat during phase transitions, PCMs can buffer temperature fluctuations and boost the backfill’s thermal storage capacity [15,17]. For example, Qi et al. [15] used numerical simulations to show that PCMs like paraffin RT27 can shrink the thermal influence radius and maintain stability. Similarly, Chen et al. [16,18] found that PCM-based grouts match the conductivity of conventional materials while significantly enhancing operational stability. In addition, a suitable phase change temperature (such as 20.4 °C) and a reasonable operating mode (such as alternating cooling and heating) can further improve system sustainability.

PCMs have inherent drawbacks, including low thermal conductivity and leakage after melting. To address these issues, researchers have modified PCMs by adding thermally conductive fillers or using shape-stabilized encapsulation [19,20,21]. In a pioneering effort, Lyne et al. [22] reinforced a commercial PCM (PureTemp29) with graphite, yielding a high-conductivity composite that remarkably boosted the GSHP system’s coefficient of performance (COP) by 112%. Parallel to this, Wan et al. [23] engineered shape-stabilized PCMs utilizing expanded perlite as a carrier and silica sol as the encapsulating agent, a method that successfully dampened temperature fluctuations within the backfill. Further advancing the field, Chen et al. [24] formulated a capric–lauric acid/expanded graphite system. This innovation not only accelerated the backfill’s heat transfer rate by 11.6% but also validated the viability of coupling fatty acid-based PCMs with expanded graphite (EG). From a design perspective, Aljabr et al. [25] utilized numerical simulations to demonstrate that optimizing PCM incorporation into grouting agents could shrink the required borehole length by approximately 7%. These projections were empirically grounded by Calviño et al. [26], whose experiments confirmed that grout mixtures infused with microencapsulated PCMs significantly elevate thermal storage capabilities. Liu et al. [27] and Zhou et al. [28] also noted that shape-stabilized PCMs show great potential for lowering soil temperature fluctuations and narrowing the underground thermal influence radius. Beyond material modification, recent inquiries have ventured into system-level integration. This includes deploying microencapsulated PCM slurries as heat transfer fluids [29] or synergizing PCMs with solar collectors and thermal storage tanks [30,31], strategies that have collectively delivered substantial gains in energy efficiency.

Despite the promising potential of PCM-modified backfill materials, critical limitations still exist in current research. First, most existing composite phase change materials (CPCMs) are based on single fatty acids or paraffin [15,16,17,18,19,22,23,24]. Single fatty acid CPCMs usually exhibit mismatched phase change temperatures with the annual soil temperature, while paraffin-based CPCMs suffer from low thermal conductivity and limited heat transfer enhancement. Studies on binary fatty acids eutectic composites blended with original sand soil (OSS) remain limited, and direct comparisons between single-component CPCMs and binary CPCMs for GSHP backfill are still insufficient. The thermal property evolution under varying moisture contents and PCM doping ratios is still not fully understood. Second, the prevailing reliance on numerical simulations or isolated performance tests fails to capture the full picture. There is a pressing need for comprehensive verification that bridges material preparation and thermal characterization with GHEs heat transfer experiments and numerical modeling. Furthermore, there is still no systematic theoretical definition of the synergistic effect between phase change heat storage capacity and thermal conductivity, especially for systems with PCM doping exceeding 10%. Establishing a clear understanding of this equilibrium is essential to guide the optimization of high-content PCM engineering applications.

Against this background, this study develops a novel composite phase change material/original sand soil (CPCM/OSS) backfill by integrating original sand soil (OSS) with a capric–myristic acid/expanded graphite (CA-MA/EG) composite. Systematic thermal characterization, including conductivity tests and differential scanning calorimetry (DSC), was performed to elucidate how moisture content and CPCM dosage govern the material’s thermophysical behavior. Furthermore, a test system for GHEs’ heat transfer performance is established and coupled with numerical simulations to dissect the heat transfer characteristics and underlying mechanisms within the CPCM/OSS system. Ultimately, this work aims to deliver a high-efficiency, stable backfill solution, providing the theoretical and technical underpinnings necessary to enhance GHEs’ performance and accelerate the engineering deployment of GSHP technology.

## 2. Results and Discussion

### 2.1. Thermal Properties of CA-MA/EG

**Figure 1:**
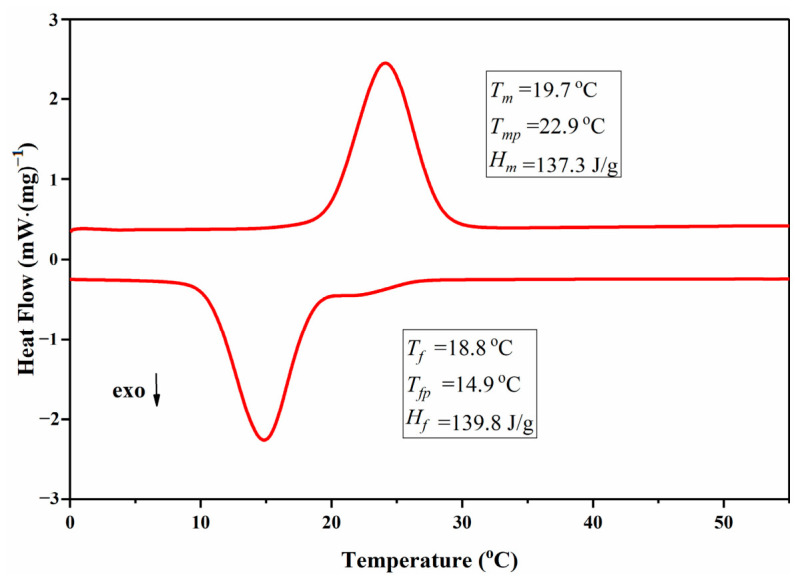
DSC curve of the CA-MA/EG CPCM (tested at 10 °C/min under N_2_ atmosphere, 0–80 °C). Subscript *m*: melting; subscript *f*: freezing; subscript *p*: peak.

The DSC curves and corresponding thermal property parameters of the CA-MA/EG CPCM are presented in Figure 1. As shown in the figure, the CA-MA/EG composite exhibits an onset melting temperature of 19.7 °C, a melting peak temperature of 22.9 °C, and a melting latent heat of 137.3 J·g^−1^. In comparison, the natural soil substrate for ground-source heat pump systems maintains a relatively stable annual temperature near 19 °C [32,33]. Overall, the phase change temperature of CA-MA/EG matches well with the typical operating temperature range of GSHP systems. Its favorable latent heat capacity further makes it a promising candidate as an efficient backfill material.

**Figure 2:**
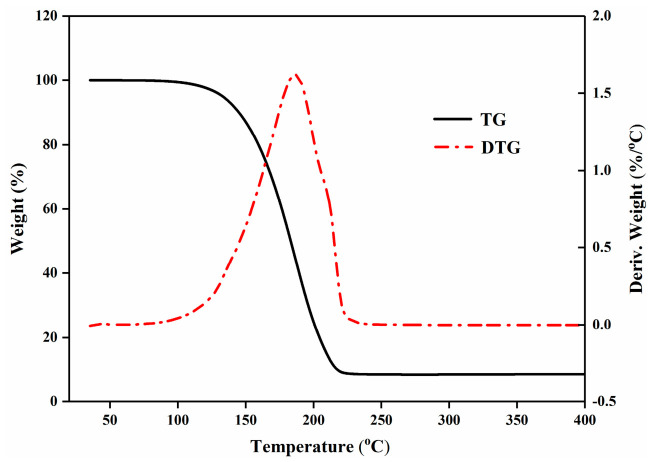
TGA curves of the CA-MA/EG CPCM.

The TGA curves of the CA-MA/EG CPCM are displayed in Figure 2. Distinct thermal decomposition behavior can be clearly observed from the TGA profiles. The CA-MA/EG CPCM begins to lose weight at approximately 107.4 °C, reaches its maximum mass loss rate at 183.4 °C, and achieves complete volatilization at around 230 °C. The residual mass is mainly attributed to inherent impurities and non-volatile expanded graphite within the composite. Notably, CA-MA/EG possesses favorable thermal stability, which makes it a promising candidate for backfill material applications for buried pipelines in GSHP systems.

### 2.2. Thermal Conductivity of CPCM/OSS

**Figure 3:**
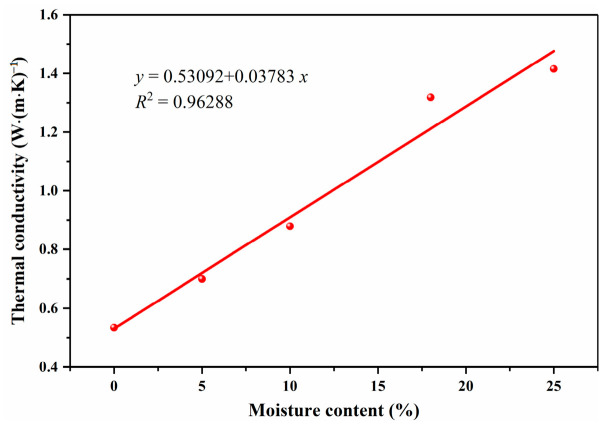
Variations in thermal conductivities of CPCM/OSS with moisture content.

The thermal conductivity (*λ*, W·(m·K)^−1^) of the CPCM/OSS energy storage backfill material is jointly affected by moisture content and CPCM dosage. Figure 3 presents the thermal conductivity variation of the CS-5 backfill sample (5% CPCM dosage) with changing moisture content. As observed from the plot, thermal conductivity shows a continuous upward trend as moisture content increases. For the dry CS-5 sample with 5% CPCM addition, the experimentally measured thermal conductivity is 0.5348 W·(m·K)^−1^. When the moisture content rises to 25%, the sand medium approaches a nearly saturated state, with the thermal conductivity reaching 1.4156 W·(m·K)^−1^. As the moisture content increases sequentially from 0% to 5%, 10%, 18%, and 25%, the thermal conductivity of CS-5 increases correspondingly from 0.5348 W·(m·K)^−1^ to 0.7003, 0.8197, 1.3187, and 1.4156 W·(m·K)^−1^, with growth rates of 30.9%, 64.4%, 146.6% and 164.7%, respectively. The corresponding linear fitting equation is established as Equation (1). Accordingly, 10% moisture content was selected as the optimal preparation condition for the fabricated CPCM/OSS backfill material.

(1) $$y=0.53092+0.03783x\;R^{2}=0.96288,$$

It should be noted that the thermal conductivity of porous sandy media generally exhibits obvious nonlinear variation near saturated moisture content due to water film percolation and pore water connectivity effects. However, within the tested moisture content range of 0–25% in this study, the measured discrete data points present an approximately linear increasing trend without obvious abrupt inflection. The fitted coefficient of determination *R*^2^ = 0.96288 indicates a high correlation and satisfactory fitting accuracy for the present experimental interval. Therefore, the linear model is adopted here to quantitatively characterize the variation trend within the current test range, which is sufficient to reflect the overall changing law and support subsequent analysis. A more complex physically based nonlinear model is unnecessary for the limited measured points and narrow variation interval in this work.

**Figure 4:**
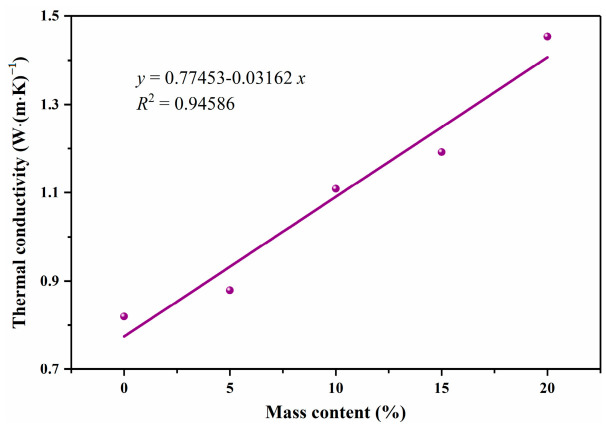
Variations in thermal conductivities of CPCM/OSS with the mass content of CPCM.

The thermal conductivity of the composite phase change material (CPCM) is superior to that of raw sand soil. Incorporating CPCM into original sand soil (OSS) can effectively improve the thermal conductivity of the composite mixture. Figure 4 illustrates the thermal conductivity evolution of CPCM/OSS composites at a fixed moisture content of 10% with varying CPCM dosage. As indicated in the figure, when the CPCM content increases from 0% to 5%, 10%, 15%, and 20%, the thermal conductivity rises accordingly from 0.8197 W·(m·K)^−1^ to 0.8793, 1.1086, 1.1923, and 1.4536 W·(m·K)^−1^, with increments of 7.3%, 35.2%, 45.5%, and 77.3%, respectively. The relevant linear fitting equation is established in Equation (2).

(2) $$y=0.77453+0.03162x\;R^{2}=0.94586,$$

### 2.3. Specific Heat Capacities and Thermal Storage Coefficient of CPCM/OSS

**Figure 5:**
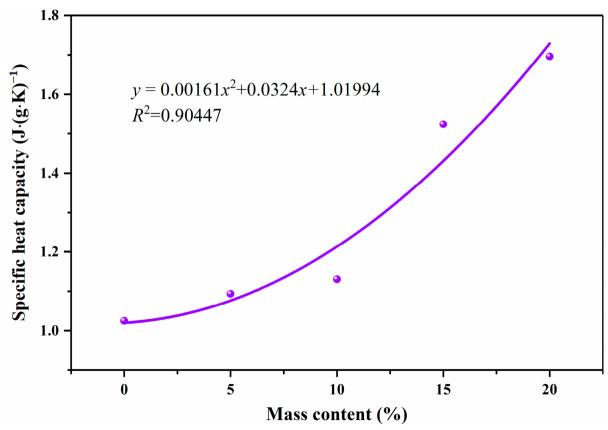
Variations in specific heat capacity of CPCM/OSS with the mass content of CPCM.

Specific heat capacity (*c*, J·(g·K)^−1^) is an essential physical parameter that evaluates the thermal storage performance of materials. Table 1 and Figure 5 summarize the measured specific heat capacity values of original sand soil and CPCM/OSS composite backfill at a fixed moisture content of 10%. Notably, the specific heat capacity of CPCM/OSS gradually rises with the increase in CPCM dosage. Quantitatively, as the CPCM content increases from 0% to 5%, 10%, 15%, and 20%, the specific heat capacity is improved by 6.7%, 10.3%, 48.8%, and 65.5%, respectively. The correlation between specific heat capacity and CPCM content conforms to a binomial fitting equation, as shown in Equation (3). CPCM/OSS possesses a higher specific heat capacity compared with pure OSS. The growth trend remains moderate when the CPCM content is within 0–10%, with only a 10.3% enhancement obtained at the 10% dosage. In comparison, a sharp rise in specific heat capacity occurs in the range of 10–20% CPCM content, reaching a maximum increment of 65.5% at 20% loading. This variation is mainly ascribed to the high latent heat characteristic of CPCM. At low CPCM dosages, the introduced CPCM provides a certain thermal storage effect, yet the overall improvement in specific heat capacity is limited. The reason is that the incorporation of CPCM reduces the bulk density of the composite and significantly increases its thermal conductivity, which to some extent offsets the thermal storage superiority of the phase-change component. With the increase in CPCM mass fraction, the thermal storage capacity of the composite gradually improves, yet its contribution remains limited at low doping levels (≤10 wt%). A noticeable enhancement in thermal storage performance only appears at relatively high CPCM contents (15–20 wt%), while the overall thermophysical improvement of the composite is still governed mainly by the enhanced thermal conductivity. Accordingly, the CPCM/OSS sample with 20% CPCM addition achieves a 65.5% higher specific heat capacity than pure OSS.

(3) $$y=0.00161x^{2}+0.0324x+1.01994\;R^{2}=0.90447,$$

**Figure 6:**
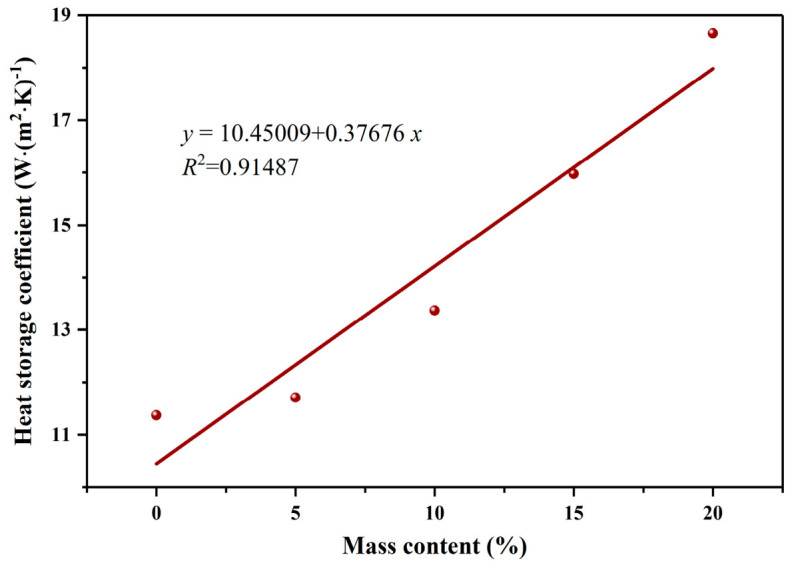
Variations in the heat storage coefficient of CPCM/OSS with the mass content of CPCM.

The thermal storage coefficient (*s*, W·(m^2^·K)^−1^) serves as a critical index for evaluating the ability of a material to resist temperature variation. It is governed by thermal conductivity, specific heat capacity, material density, and the fluctuation period of heat flux. A higher thermal storage coefficient corresponds to superior thermal stability. The thermal storage coefficient can be calculated according to Equation (4) [34]. Table 2 and Figure 6 present the calculated results for the CPCM/OSS composites. It is evident that the thermal storage coefficient rises steadily with the increase in CPCM dosage. As the CPCM content increases from 0% to 5%, 10%, 15%, and 20%, the thermal storage coefficient increases by 3.0%, 17.6%, 40.5%, and 64.1%, respectively. The relationship between the two parameters is well fitted by a linear equation, as described in Equation (5). Compared with pure OSS, CPCM/OSS-modified sand achieves a remarkable enhancement in thermal storage coefficient. This improvement originates from the simultaneous increase in specific heat capacity and thermal conductivity with rising CPCM loading, which collectively contributes to a distinct elevation in the thermal storage coefficient of the composite.

(4) $$s=\sqrt{\frac{2\pi c\rho \lambda }{Z}},$$

where *Z* is the period of temperature fluctuation, (s, typically taken as 24 h = 86,400 s for calculation)

(5) $$y=10.45009+0.37676x\;R^{2}=0.91487,$$

From the perspective of single thermophysical performance, thermal conductivity, specific heat capacity, and thermal storage coefficient all increase monotonically with CPCM dosage, and the 20 wt% sample presents the highest comprehensive thermal parameters. However, the optimal dosage for practical engineering backfill application cannot be determined merely by a single thermal performance. It requires a comprehensive trade-off among the thermal enhancement effect, material economic cost, mechanical structural stability, and on-site construction workability.

### 2.4. Phase Change Temperature and Latent Heat of CPCM/OSS

**Figure 7:**
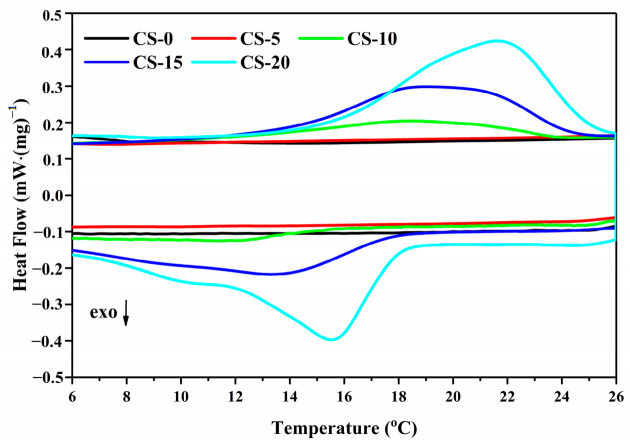
DSC curves of CPCM/OSS backfill material.

Figure 7 shows the DSC curves of the CPCM/OSS backfill composites. For the CS-0 sample, the heating and cooling profiles are nearly linear, without obvious endothermic or exothermic peaks. This indicates that pristine sand soil possesses no thermal energy storage capacity and behaves as a material with stable and constant thermophysical properties. In theory, weak endothermic and exothermic signals should be detectable for CS-5 with 5% CPCM incorporation. Nevertheless, the low CPCM dosage, combined with inevitable experimental sampling errors, results in a DSC profile almost identical to that of CS-0, showing an approximately linear trend without distinguishable phase-change peaks. As the CPCM loading further increases, CS-10, CS-15, and CS-20 all exhibit well-defined endothermic and exothermic peaks, along with a gradual expansion of the phase-change region. Meanwhile, both the phase-change onset point and phase-change temperature shift toward higher values. The characteristic phase-change temperatures and latent heat values of all samples are summarized in Table 3.

It can be seen from Table 3 that the melting temperature of CPCM/OSS composites is obviously lower than the freezing temperature. In particular, the melting point of CS-10 decreases remarkably from 19.7 °C of pure CA-MA/EG to 10.4 °C, with a substantial temperature shift of nearly 9 °C. Such a large deviation cannot be merely attributed to conventional pore confinement and signal-to-noise interference; it arises from multiple coupled mechanisms. The microporous structure of OSS imposes strong spatial confinement on PCM molecules to restrict molecular chain rearrangement and lattice ordering. Interfacial interactions between quartz sand and fatty acid molecules further disrupt the regular eutectic crystal structure. At the low 10 wt% dosage, fine isolated CPCM dispersion induces grain refinement, which lowers the phase transition temperature following the melting point size effect. Moreover, the large heat capacity of the OSS matrix causes baseline drift during DSC rapid heating, overlapping with the weak endothermic peak of low-content CPCM and further reducing the apparent phase change temperature. During crystallization, pore restriction on nucleation and growth is much weaker, resulting in a higher freezing temperature. The low CPCM fraction also produces a weak DSC signal, and internal temperature gradients together with test baseline fluctuations lead to reasonable melting–freezing hysteresis. Importantly, 10.4 °C is only the apparent phase transition temperature under DSC fast heating. In practical GSHP engineering, underground soil operates under slow quasi-steady thermal cycling. Under such natural gradual temperature variation, the actual phase-transition interval of CPCM broadens and rebounds toward 19.7 °C, still matching the annual soil temperature range. The performance improvement of CS-10 is dominated by enhanced thermal conductivity, while its latent heat mainly serves temperature buffering and fluctuation suppression under intermittent operation. Despite the apparent low-temperature shift of the DSC peak, the composite still maintains reliable thermal regulation capability and good engineering adaptability.

The low latent heat of all CPCM/OSS samples stems from the low CPCM doping ratio (5–20 wt%) and the strong dilution effect of the OSS matrix. The pure CA-MA/EG has a latent heat of 137.3 J·g^−1^, while the effective utilization ratio of CPCM in CS-10 is only about 3.77%, insufficient to dominate thermal storage performance. Pore confinement and partial encapsulation of CPCM particles by sand grains further suppress the full release of phase-change enthalpy. Since OSS has no intrinsic phase-change activity, the composite latent heat solely comes from the dispersed CA-MA/EG component. Small DSC sampling mass and particle wrapping also weaken the measured latent heat signal, which is a typical feature of low-concentration phase-change backfill materials. According to the literature [35,36], 5 J·g^−1^ is recognized as the threshold for effective thermal storage of building materials. The latent heat of CS-10 reaches 5.18 J·g^−1^, satisfying this threshold and endowing the composite with reliable temperature-buffering ability. Nevertheless, its contribution to heat exchange enhancement remains limited and cannot dominate thermal performance improvement. Although higher CPCM dosage (15–20 wt%) can further improve thermophysical properties, the marginal performance gain gradually decreases, accompanied by obvious disadvantages of sharp cost growth, reduced compactness and mechanical strength of composite backfill, as well as increased segregation risk during on-site mixing and backfilling construction. By comparison, 5 wt% CPCM only achieves limited thermal improvement and cannot reach the effective thermal storage threshold. The 10 wt% formulation balances satisfactory thermal conductivity enhancement, qualified latent heat storage capacity, controllable material cost, good mechanical stability, and construction adaptability. Therefore, the 10 wt% CPCM dosage is comprehensively selected as the optimal formulation through multi-index comprehensive optimization rather than single thermal performance optimization.

### 2.5. Heat Exchange Performance Test Results Analysis

**Figure 8:**
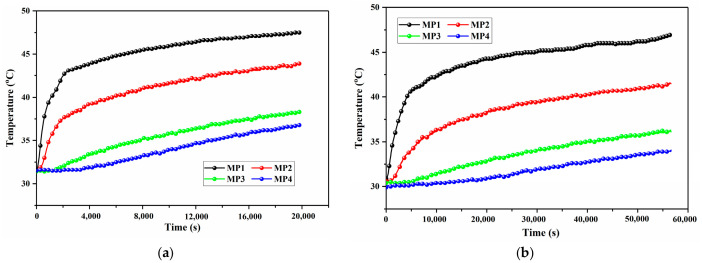
Temperature change of backfill areas and around soil at two backfilling modes: (**a**) CPCM/OSS backfilling; (**b**) OSS backfilling.

The circulating water temperature inside the ground heat exchanger was kept constant at 60 °C. Two backfill working conditions were compared in the test: pure OSS and CPCM/OSS composite doped with 10 wt% CPCM. Figure 8 illustrates the temporal evolution of soil temperature distribution in both the backfill region and the surrounding soil layer of the ground heat exchanger under these two conditions.

#### 2.5.1. Spatiotemporal Distribution Characteristics of Temperature Field

Comparison between Figure 8a,b shows distinct differences in the spatiotemporal temperature evolution under the two backfill schemes. The temperature discrepancy between the two backfill schemes originates mainly from the distinct thermal conductivity of CPCM/OSS, while the latent heat effect plays only an auxiliary role. The performance improvement of the composite backfill is primarily governed by its enhanced thermal conductivity, which accelerates radial heat migration and alleviates heat accumulation near the borehole. Owing to the extremely low effective latent heat utilization (only 3.77%) at the optimal 10 wt% CPCM dosage, the phase-change component can only provide a mild temperature buffering effect, suppressing excessive temperature rise during operation and slowing soil temperature drop during intermittent shutdown. It does not dominate the overall heat transfer enhancement of the ground heat exchanger. Temporally, the whole heat transfer process can be divided into three evolutionary stages controlled primarily by heat conduction. In the initial stage, the temperature rise rate at the backfill core is similar for both CPCM/OSS and pure OSS, and the CPCM only functions as a weak thermal buffer to slow outward heat diffusion. In the middle stage, the superior thermal conductivity of CPCM/OSS becomes the controlling factor, significantly accelerating heat transfer to the far-field soil layer and enlarging the thermal influence radius. In the later stable diffusion stage, the overall soil temperature maintained by CPCM/OSS remains higher owing to persistent efficient heat conduction, which mitigates thermal saturation and sustains long-term stable heat exchange. At 15,000 s, the average soil temperature increases by approximately 5 °C for CPCM/OSS, but only 2.2–2.5 °C for OSS, with a difference of 2.5–2.8 °C. This indicates that CPCM/OSS effectively regulates the soil thermal environment, slows the attenuation of the temperature difference between GHEs and soil, and supports long-term stable heat exchange.

Spatially, the temperature gradient decreases from approximately 0.8 °C·cm^−1^ for OSS to 0.5 °C·cm^−1^ for CPCM/OSS in the radial direction. The radial heat diffusion range is expanded significantly, indicating that CPCM/OSS alleviates the thermal conductivity bottleneck of conventional backfill materials and improves overall heat transfer efficiency.

#### 2.5.2. Quantitative Comparison of Temperature Responses at Key Measurement Points

The temperature data at key monitoring points are quantitatively compared in Table 4, which are obtained from the heat transfer experiment results presented in Figure 8. At this moment, the heat transfer process tends to be stable, and the temperature difference between the CPCM/OSS and OSS backfill systems is sufficiently significant to reflect the heat transfer enhancement effect. The results show that temperature increments are more significant at distant points (MP3, MP4), which is of great engineering importance. Conventional OSS backfill tends to cause heat accumulation near the GHEs, leading to the thermal short-circuit effect and degraded heat exchange efficiency. In contrast, CPCM/OSS backfill effectively transfers heat to distant soil layers and slows down soil thermal saturation. Given the typical GHE spacing of 3–5 m in engineering applications, the 7.2 °C temperature rise at MP4 indicates that heat can diffuse to a wider range, reducing thermal interference between adjacent boreholes. Moreover, CPCM/OSS can store heat during system operation and release it slowly during shutdown periods. This avoids sharp temperature drops of the surrounding soil and maintains a favorable temperature difference for the next operation cycle, making it well adapted to the intermittent operation mode of GSHP systems.

In summary, the temperature evolution results verify the superior heat transfer performance of CPCM/OSS and reveal that the overall heat transfer and temperature field improvement is primarily governed by thermal conductivity enhancement, while the qualified latent heat that reaches the application threshold acts as an indispensable auxiliary role in stabilizing soil temperature and adapting to intermittent operation.

### 2.6. Numerical Simulation Results and Analysis

Numerical simulations were performed to examine the heat transfer characteristics of a U-tube GHE under intermittent summer operating conditions. Two backfill materials were evaluated: conventional OSS and CPCM/OSS composite doped with 10 wt% CPCM. Simulations covered three consecutive days, with a daily cycle consisting of 10 h of operation followed by 14 h of shutdown. Identical boundary and operating conditions were applied to both systems to ensure a fair comparison of heat transfer performance.

**Figure 9:**
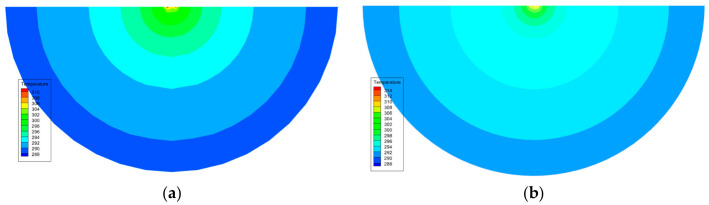
Horizontal temperature distribution contour at 5 m depth: (**a**) OSS backfilling; (**b**) CPCM/OSS backfilling.

**Figure 10:**
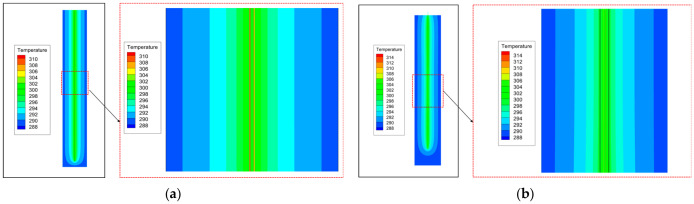
Vertical temperature distribution contour at 5 m depth: (**a**) OSS backfilling; (**b**) CPCM/OSS backfilling.

The horizontal and vertical temperature distributions for the two backfill systems are presented in Figure 9 and Figure 10, respectively. The temperature field of the CPCM/OSS backfill system is consistently higher than that of the OSS system. This difference is mainly attributed to the higher thermal conductivity of CPCM/OSS (1.1086 W·(m·K)^−1^) relative to OSS (0.8197 W·(m·K)^−1^). The improved thermal conductivity effectively accelerates heat transfer from the GHEs to the surrounding soil.

#### 2.6.1. Horizontal Temperature Field Characteristics and Heat Diffusion Mechanism

Figure 9 shows the horizontal temperature contours at 5 m depth for the two backfill systems. The temperature diffusion patterns differ significantly, reflecting the strong influence of backfill properties on heat transfer efficiency. In the OSS system (Figure 9a), the high-temperature region (≥308 K) is limited to a radius of 0.2 m around the U-tube. Temperature drops quickly beyond this zone: it falls below 302 K at 0.5 m and approaches the initial soil temperature of 298 K at 1.0 m. This narrow high-temperature zone and rapid thermal attenuation reveal that the low thermal conductivity of OSS restricts heat diffusion and causes severe heat accumulation near the GHEs. Long-term operation may induce a thermal short-circuit effect, which reduces the GHE–soil temperature difference and degrades heat exchange performance. By contrast, the high-temperature zone expands noticeably in the CPCM/OSS system (Figure 9b). The temperature remains above 304 K within a radial distance of 0.8 m and is 2–3 K higher than that of the OSS system at 1.5 m. The radial temperature gradient decreases from 0.8 K·cm^−1^ for OSS to 0.5 K·cm^−1^ for CPCM/OSS, indicating a more uniform temperature distribution. This improved heat diffusion is predominantly attributed to the enhanced thermal conductivity of CPCM/OSS, which dominates the expansion of the thermal influence range and the homogenization of the temperature field. The latent heat of CA-MA/EG plays only a secondary auxiliary role in mildly restraining local overheating, with negligible contribution to the overall heat exchange capacity improvement. In engineering practice, the expanded heat influence radius helps reduce thermal interference between adjacent boreholes (typically spaced 3–5 m apart) and alleviates soil thermal saturation.

#### 2.6.2. Vertical Temperature Field Uniformity and Intermittent Operation Adaptability

Figure 10 presents the vertical temperature distributions of the two backfill systems, which directly determine the overall heat exchange performance of GHEs. For the pure OSS backfill system (Figure 10a), significant vertical temperature stratification is observed. The temperature in the shallow layer (0–10 m) is generally below 302 K, whereas that in the middle–deep layer (20–30 m) reaches up to 306 K, with a maximum vertical temperature difference of 4 K. This inhomogeneity arises from the low thermal conductivity of OSS, which impedes vertical heat transfer and causes uneven heat accumulation. In contrast, the CPCM/OSS backfill system (Figure 10b) exhibits excellent vertical temperature uniformity. Over the entire burial depth of 0–50 m, the temperature fluctuates within a narrow range of 304–306 K, corresponding to a total difference of only 2 K. This improvement in vertical temperature uniformity is primarily governed by the superior thermal conductivity of the CPCM/OSS composite backfill. The enhanced thermal conductivity facilitates efficient and uniform vertical heat propagation throughout the soil layer and effectively eliminates localized heat accumulation around the ground heat exchanger. Meanwhile, the phase-change latent heat of CA-MA/EG has reached the applicable threshold for building thermal storage materials. Although the effective utilization ratio is only 3.77%, it still performs reliable dynamic thermal regulation. Consequently, the temperature of the CPCM/OSS system decreases by only 1–1.5 K during the 14 h intermittent period. This allows the system to quickly restore high-efficiency heat exchange upon restart, demonstrating superior adaptability to the typical “daytime operation, nighttime shutdown” intermittent mode of ground-source heat pump systems.

#### 2.6.3. Quantitative Analysis of Heat Exchange Capacity

**Figure 11:**
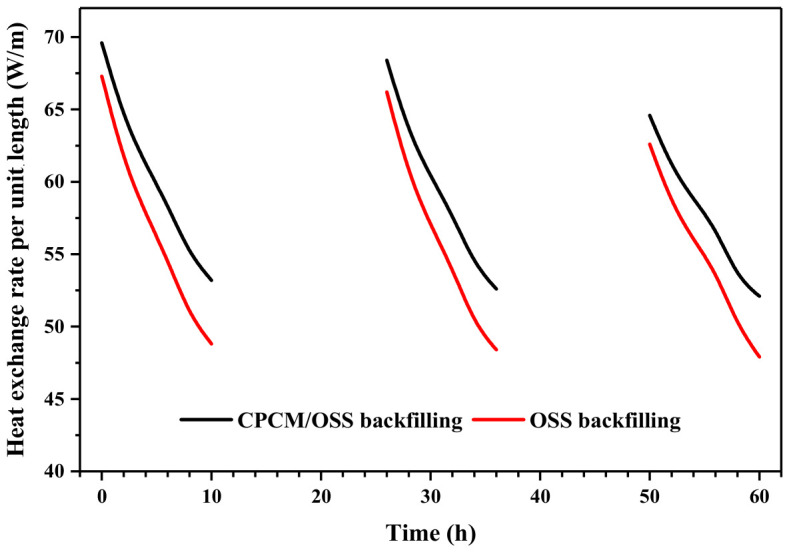
The temporal variation of the heat exchange rate per unit length.

The heat exchange rate per unit length (*q_l_*, W·m^−1^) of the GHEs was calculated using Equation (6) [8,11], based on the inlet and outlet water temperatures from simulations. The time-dependent variation of *q_l_* for both backfill systems is plotted in Figure 11.

(6) $$q_{l}=\frac{c\rho v(t_{o}-t_{i})\;}{l},$$

where *q_l_* is the heat exchange rate per unit length of the ground heat exchanger, W·m^−1^; *c* is the specific heat capacity of the circulating fluid in the tube, J·(g·K)^−1^; *ρ* is the density of water inside the ground heat exchanger, kg·m^−3^; *v* is the velocity of water inside the ground heat exchanger, m·s^−1^; *t_o_* and *t_i_* are the outlet and inlet water temperatures inside the ground heat exchanger, respectively, °C.

Over three days of intermittent operation, the heat exchange rate of both systems declines slightly. The attenuation reaches 25% for CPCM/OSS and 28% for OSS, relative to the initial values. This trend arises from the thermal accumulation effect: continuous heat input raises the soil temperature and reduces the GHEs–soil temperature difference, weakening the heat exchange driving force. Notably, the CPCM/OSS system shows a slower attenuation rate, indicating better mitigation of thermal accumulation. After three days, the unit-length heat exchange rate is 52.1 W/m for CPCM/OSS and 47.9 W/m for OSS, corresponding to an 8.8% improvement with the composite backfill. It is noteworthy that the thermal conductivity of the 10 wt% CPCM composite is increased by about 35.2%, while the overall heat exchange rate of the borehole only presents a modest improvement of 8.8%. This obvious mismatch indicates that the system heat transfer performance is not solely controlled by the backfill thermal conductivity. The total borehole thermal resistance consists of multiple series thermal resistance components, including pipe wall conduction, internal fluid convective resistance, and undisturbed soil thermal resistance. These parts occupy a large proportion of the total heat transfer process and become the dominant limiting factors. Therefore, even if the backfill thermal conductivity is significantly enhanced, the overall heat transfer improvement of the ground heat exchanger is still constrained, resulting in a relatively low system-level promotion rate. This quantitative result confirms that the 8.8% enhancement of heat exchange capacity is mainly driven by the significant improvement in thermal conductivity. Although the latent heat of the optimal CS-10 formulation meets the thermal storage threshold and effectively optimizes the system adaptability to intermittent operating conditions, its low utilization rate leads to a secondary rather than dominant contribution to the overall heat exchange promotion. It also agrees well with the laboratory tests in Section 2.5, where CPCM/OSS increased the average soil temperature by approximately 5 °C, providing consistent cross-validation between experiment and simulation.

#### 2.6.4. Parametric Sensitivity Analysis

To verify the robustness and prediction reliability of the established numerical model, a parametric sensitivity analysis was conducted on four key influencing factors, including the thermal conductivity and specific heat capacity of CPCM/OSS backfill, initial soil temperature, and inlet water flow velocity. Each parameter was varied within a reasonable engineering range around the baseline value, and its influence on soil temperature response and heat exchange performance was analyzed qualitatively. The results indicate that the thermal conductivity of backfill material exerts the most prominent influence on the heat transfer performance of ground heat exchangers. The specific heat capacity and initial soil temperature show a moderate sensitivity level to the thermal response. In contrast, the inlet flow velocity presents a relatively weak impact within the practical operating range of ground source heat pump systems. Overall, the numerical results are not obviously affected by reasonable fluctuations of the above key physical and operational parameters. The calculation of the numerical model is robust, and the optimal 10 wt% CPCM doping ratio determined in this study is reliable and applicable for practical engineering conditions.

In summary, numerical simulation results confirm that the CPCM/OSS composite backfill material effectively improves heat transfer efficiency owing to its high thermal conductivity. It also optimizes both horizontal and vertical temperature field distributions and enhances adaptability to intermittent operating conditions. These favorable features jointly promote the performance and operational reliability of GSHP systems, offering solid technical support for the engineering application of CPCM/OSS backfill materials.

In this study, multiple quantitative validation metrics are adopted to verify the reliability of experimental and numerical results. The maximum relative deviation of repeated thermophysical property tests is within 3%, while the inherent measurement errors of DSC, TGA, and thermal conductivity tests are controlled within ±4% and ±3%, respectively. All thermocouples were calibrated in advance with a temperature measurement uncertainty of ±0.1 °C. Mesh independence verification was implemented to eliminate grid-induced numerical deviation. The simulation results agree well with experimental temperature variation trends, and the optimal CPCM/OSS backfill achieves a quantitative heat transfer enhancement of 8.8% compared with pure OSS. These error ranges, test repeatability, sensor accuracy, and performance enhancement rate jointly serve as reliable quantitative validation metrics, confirming the credibility of the present research outcomes.

The numerical results are further quantitatively validated against experimental measurements. The simulated heat exchange capacity enhancement reaches 8.8%, which is exactly consistent with the experimental value. Meanwhile, the simulated soil temperature rise difference between CPCM/OSS and OSS backfill agrees well with the experimental range of 2.5–2.8 °C in both variation trend and magnitude. The consistent enhancement rate and temperature response range confirm the rationality and accuracy of the established numerical model. Accordingly, the simulation results in Figure 9, Figure 10 and Figure 11 possess reliable predictive capability rather than merely qualitative description.

### 2.7. Comparison with the Literature CPCMs

As shown in Table 5, most reported PCM-based backfill materials for GSHP systems are based on single fatty acids or paraffin [15,16,17,18,22,23,24]. Single fatty acid-based backfills often suffer from mismatched phase change temperatures with the annual soil temperature (~19 °C). Paraffin-based backfills generally show relatively low thermal conductivity, limiting further improvement of heat transfer efficiency.

In contrast, the binary fatty acid CPCM/OSS backfill prepared in this work presents a phase change temperature of 19.7 °C, which matches well with the in situ soil temperature. The thermal conductivity reaches 1.1086 W·(m·K)^−1^, significantly higher than that of most single-component PCM backfills. The heat exchange capacity per linear meter is enhanced by 8.8% compared with pure OSS backfill.

These results confirm that the proposed binary CPCM/OSS backfill exhibits obvious advantages over single fatty acid or paraffin-based backfills in temperature matching, thermal conductivity, and heat transfer performance.

## 3. Materials and Methods

### 3.1. Materials

The raw materials used in this experiment fall into four categories: original sandy soil taken from ground source heat pump backfill wells, capric acid (CA), myristic acid (MA), and expandable graphite. The CA-MA/EG composite phase change material was fabricated by combining the two fatty acids with expanded graphite. Notably, expanded graphite was obtained via thermal expansion of expandable graphite. Two backfill specimens were prepared in this study: original sandy soil alone and a composite mixture of original sandy soil and CA-MA/EG, designated as CPCM/OSS.

OSS was extracted from the backfill well of a GSHP project. Its key physical and thermal properties were characterized via standard geotechnical and thermal tests, revealing that quartz (SiO_2_) was the main chemical component, with minor contributions from feldspar minerals. Capric acid and myristic acid were purchased from Shanghai Zhanyun Chemical Co., Ltd. (Shanghai, China), both with a purity of ≥98.5%. Expandable graphite was provided by Qingdao Hengrunda Graphite Products Co., Ltd. (Qingdao, China). Expanded graphite, which acted as the supporting matrix for the CPCM, was prepared by high-temperature thermal expansion of the as-received expandable graphite. The key physical and thermal properties of all raw materials are summarized in Table 6.

The physical parameters of the original sand soil were measured experimentally in this study and referenced to the national standard [33]. The thermophysical properties of capric acid, myristic acid, and expandable graphite were obtained from the manufacturer product manuals and the published literature [37,38].

### 3.2. Preparation of CA-MA/EG

**Figure 12:**
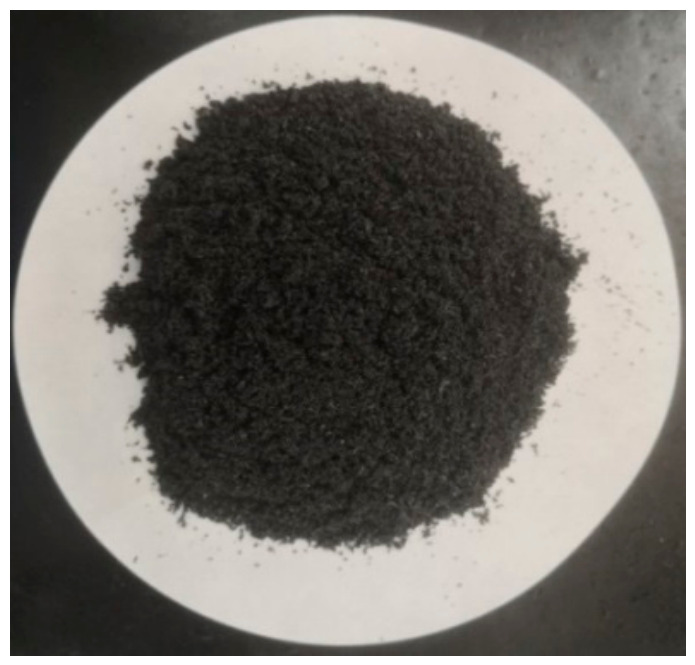
CA-MA/EG CPCM.

Capric acid and myristic acid were weighed at the predetermined mass ratio using an electronic balance, transferred into a beaker, and mixed thoroughly with a glass rod to form a preliminary blend. The beaker was then placed in a 70 °C constant-temperature water bath, where the mixture was stirred continuously at 300 r/min for 30 min until a homogeneous, transparent melt formed. The resulting melt was poured into a clean container and cooled at 4 °C in a dust-free environment to yield the CA-MA eutectic mixture, which was then sealed and stored in a desiccator. Separately, expandable graphite was subjected to high-temperature thermal expansion in a muffle furnace: the temperature was raised to 800–850 °C at 10–15 °C·min^−1^ and held for 60–90 s to achieve rapid expansion. After cooling naturally to room temperature inside the furnace, the light, porous, worm-like expanded graphite (EG) was collected, sealed, and stored for later use. The pre-prepared CA-MA eutectic mixture and EG were combined in a beaker at the predetermined mass ratio. The mixture was heated in a 70 °C water bath with continuous stirring at 300 r·min^−1^. Once the eutectic mixture had fully melted, stirring was maintained for an additional 40–60 min to ensure uniform adsorption of the molten CA-MA into the porous structure of EG. Afterward, the blend was left to cool at room temperature in a dust-free environment, yielding the CA-MA/EG composite phase change material (CPCM). The final composite was sealed and stored in a light-protected desiccator for subsequent experiments. The binary CA-MA eutectic mixture and CA-MA/EG CPCM were successfully fabricated in this work. The mass ratio of CA to MA was determined as 72.2:27.8, and expanded graphite occupied 92.2% of the composite matrix [37,38]. The morphologies of the prepared CA-MA/EG samples are displayed in Figure 12.

### 3.3. Preparation of CPCM/OSS

A portion of shallow-layer original sandy soil was first sieved to remove large gravel and impurities, then weighed and dried thoroughly in a vacuum drying oven to eliminate residual free moisture before use. The fully dried sandy soil was cooled naturally to room temperature and sealed for subsequent batching. A preset mass of the prepared CA-MA/EG composite was weighed according to the designed mass fraction and uniformly blended with the pretreated dry sandy soil. An appropriate amount of deionized water was gradually added to the mixture, followed by manual stirring to achieve homogeneous component dispersion. Thereafter, the mixed raw materials were placed in a sealed container for static placement to ensure sufficient moisture infiltration and homogenization inside the mixture. In this way, the CPCM/OSS phase-change energy-storage backfill material was fabricated and hermetically sealed for subsequent thermophysical and heat transfer tests.

The optimal moisture content of backfill sandy soil ranges from 8% to 12%. In this study, the moisture content of the prepared CPCM/OSS backfill was precisely controlled at 10%. The dry density of raw backfill sandy soil was measured to be 2.063 g·cm^−3^, with a minimum compaction coefficient requirement of 0.95. Accordingly, the target density of the as-fabricated CPCM/OSS backfill material was set at 1.961 g·cm^−3^.

**Figure 13:**
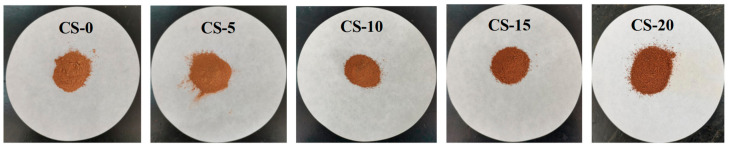
CPCM/OSS backfill materials.

To explore the influence of CA-MA/EG CPCM dosage on the comprehensive performance of CPCM/OSS backfill material, a set of specimens with different CA-MA/EG mass fractions was fabricated. A control group composed of pure original sandy soil without CPCM addition was prepared for baseline comparison. Meanwhile, four CPCM/OSS mixtures were formulated with CA-MA/EG mass fractions of 5%, 10%, 15%, and 20% by total mass. All samples were labeled CS-0, CS-5, CS-10, CS-15, and CS-20 in sequence, as presented in Figure 13.

### 3.4. Test System for Heat Transfer Performance

**Figure 14:**
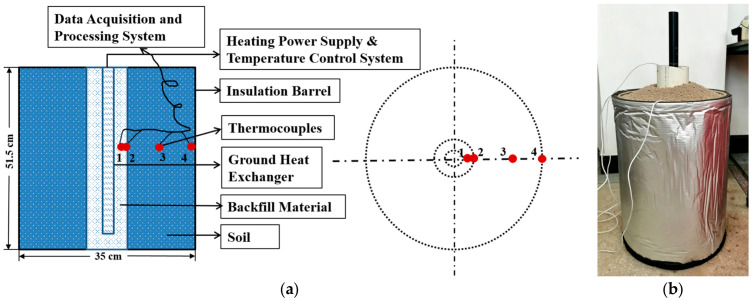
Schematic and actual photograph of the vertical U-tube heat transfer experimental setup: (**a**) Schematic diagram of the test system; (**b**) Actual photograph of the experimental setup.

The experimental system mainly comprised an insulated cylinder, a simulated heat source, thermocouple temperature sensors, a data acquisition and processing unit, and backfill material specimens. Two types of backfill were tested for comparison: pure OSS and 10 wt% CPCM-doped CPCM/OSS. The insulation cylinder had an inner diameter of 35 cm and a height of 51.5 cm, and its structural schematic is shown in Figure 14. Figure 14a shows the schematic diagram of the test system, while Figure 14b is the actual photograph. As depicted in the figure, the prepared backfill samples were densely filled into a circular tube with a diameter of 10 cm, which was placed at the central position of the cylinder. A DN32 GSHP borehole heat exchanger was inserted along the central axis of the sample column, and hot water circulated inside the exchanger to serve as the simulated heat source. A constant-temperature heater was used to keep the water temperature stable at 60 °C. It is well known that the circulating fluid in practical ground-coupled heat pump systems typically operates within 5–30 °C throughout the year. In this laboratory test, a constant inlet temperature of 60 °C was deliberately adopted as an accelerated thermal excitation condition, rather than a simulation of actual steady engineering operation. This setting can rapidly form a significant temperature gradient around the borehole and effectively highlight the difference in heat transfer characteristics between ordinary sand backfill and CPCM composite backfill for comparative analysis.

The annular gap outside the central circular tube was fully filled with raw OSS. On the horizontal cross-section at the cylinder mid-height, temperature monitoring points were arranged radially outward from the heated borehole exchanger. Measurement Point 1 (MP1) was located at the sample center; MP2 lay at the interface between the CPCM/OSS sample and surrounding OSS; MP4 was set at the inner wall of the insulation cylinder; MP3 was arranged midway between MP2 and MP4.

After layout completion, the central circular tube was removed, and the surrounding OSS was compacted to achieve close contact with the backfill specimen. The heating system was switched on to rapidly raise the water temperature inside the borehole heat exchanger to 60 °C. Meanwhile, the data acquisition instrument was started to record temperature signals at 10 s intervals for automatic storage. The experiment was terminated once a noticeable temperature increase appeared at the outermost measuring point.

### 3.5. Characterization

A differential scanning calorimeter (DSC, NETZSCH 214 Polyma, Selb, Germany) was used to test the phase change characteristics of the prepared materials, including melting temperature *T_m_*, freezing temperature *T_f_*, as well as melting latent heat *H_m_* and freezing latent heat *H_f_*. The DSC tests were performed at a constant heating rate of 10 °C·min^−1^, with the temperature ranging from 0 °C to 80 °C. According to the instrument parameters, the measurement accuracy was ±0.1 °C for phase change temperature and ±4% for latent heat.

Thermogravimetric analysis (TGA, TA TGA5000IR, New Castle, DE, USA) was adopted to assess the thermal resistance and thermal stability of the samples. Temperature calibration was conducted using indium oxide (In_2_O_3_), tin (Sn), and zinc (Zn), while balance sensitivity was calibrated with standard weights. The TGA measurement was carried out under a nitrogen atmosphere at a heating rate of 10 °C·min^−1^. The resulting temperature deviation was controlled within ±2 °C, and the mass measurement error was limited to ±0.05%. Before formal testing, blank baseline experiments with empty Al_2_O_3_ crucibles were completed under the same experimental conditions. All sample curves were baseline-corrected using the built-in instrument software to eliminate background interference.

A thermal conductivity tester (DRE-III, Xiangtan Xiangyi Instrument Co., Ltd., Xiangtan, China) was used to measure the thermal conductivity and thermal storage coefficient of the specimens. Each sample was tested in triplicate, and the averaged values were adopted for further data analysis. Before measurement, specimens were placed into a special mold, and the test probe was tightly attached to the sample surface in strict accordance with the instrument operating procedure. A hydraulic compression device was applied to compact the specimens to the predetermined target density. Real-time pressure monitoring was conducted to guarantee uniform stress distribution and avoid sample cracking or deformation caused by over-compression. Once the sample density reached the required standard, the instrument was turned on. Key experimental parameters, including the temperature control range and holding time, were set according to the technical specifications of the DRE tester. The steady-state test was then started, and experimental data were automatically recorded and output by the device. As stated in the instrument calibration certificate, the maximum allowable measurement error was within 3%, which fully met the accuracy demands for thermal performance characterization of backfill materials.

### 3.6. Uncertainty Analysis

Experimental uncertainties in this study mainly originate from specimen preparation, instrument measurement accuracy, manual operation, and numerical model simplification.

For DSC and TGA tests, the temperature measurement uncertainty is within ±2 °C, and the relative error of latent heat and mass loss evaluation is less than ±4%, which meets the standard characterization requirement of phase-change composite materials. For thermal conductivity and specific heat capacity measurements, each specimen was tested in triplicate, and the average value was adopted. The maximum relative deviation of repeated tests is within 3%, and the inherent instrument error is controlled below 3%.

In the heat transfer experiment, all thermocouples were calibrated in advance, with a temperature measurement uncertainty of ±0.1 °C. The systematic error caused by manual filling, compaction, and layout of monitoring points is within a reasonable range. For numerical simulation, mesh independence verification and reasonable physical assumptions were adopted to reduce numerical calculation deviation.

Overall, all experimental data and simulation results in this work are within acceptable uncertainty limits, which verifies the reliability and rationality of the research conclusions.

### 3.7. Application Simulation of Phase Change Backfill Materials

#### 3.7.1. Modeling Assumptions

To simplify the heat transfer analysis of the ground heat exchanger, the following modeling assumptions were adopted.

(1) The backfill material and native soil are both treated as homogeneous and isotropic porous media, with constant thermophysical properties throughout the entire heat transfer process.
(2) The circulating fluid in the U-tube is assumed incompressible. Its thermophysical parameters, such as thermal conductivity, specific heat capacity, and density, remain constant, while the heat transfer effect induced by fluid viscous dissipation is neglected.
(3) Contact thermal resistance between the U-tube wall and backfill, as well as that between backfill and surrounding soil, is ignored.
(4) Vertical heat transfer in the soil is neglected; only horizontal heat conduction within the soil domain is considered.
(5) Heat transfer among the ground heat exchanger, backfill material, and surrounding soil is simplified to pure heat conduction [39].
(6) The flow inside the tube is regarded as turbulent, given that the corresponding Reynolds number exceeds 3000 [40].

#### 3.7.2. Physical Model

**Figure 15:**
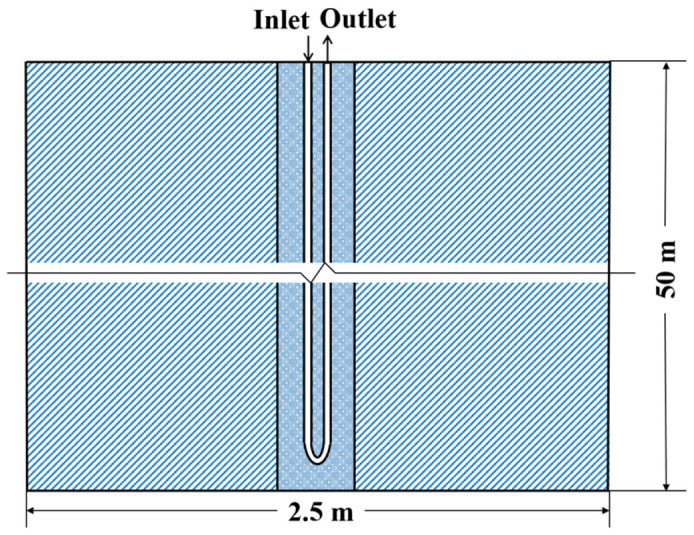
Physical model of vertical single U-Tube ground.

The geometric parameters of the physical model are presented in Table 7. Based on the abovementioned assumptions, the established physical model is schematically illustrated in Figure 15.

#### 3.7.3. Mathematical Model

To characterize the coupled heat transfer, phase change, and fluid flow in the PCM-incorporated backfill zone, the mass, energy, and momentum conservation laws are employed, along with the porous-medium assumption for PCM. The governing equation of the numerical model is formulated as Equation (7) [39,40].

(7) $$\frac{\partial \rho }{\partial t}+\nabla \cdot \left(\rho \vec{v}\right)=0,$$

where *ρ* is the density of the grouting material, kg·m^−3^; *t* is time, s; $\vec{v}$ is the velocity vector, m·s.

Next, the energy conservation equation is established based on the enthalpy model. This method is widely adopted for phase change problems because it eliminates the need to explicitly track the moving phase interface. The equation simultaneously considers sensible heat induced by temperature variation and latent heat generated during phase transition, as presented in Equation (8) [39,40].

(8) $$\frac{\partial \rho }{\partial t}\left(\rho H\right)+\nabla \cdot \left(\rho \vec{v}H\right)=\nabla \cdot \left(k\nabla T\right)+S_{e},$$

where *S_e_* is the heat source term, W·m^−3^; *H* is the enthalpy of the grouting material, J·g^−1^. The enthalpy *H* is defined as Equation (9) [39,40].

(9) $$H=h_{ref}+\int_{T_{ref}}^{T}c_{p}dT+\beta L,$$

where *h_ref_* is the reference enthalpy, J·g^−1^; *L* is the latent heat of phase change of the PCM, J·g^−1^; *c_p_* is the constant-pressure specific heat capacity, J·(g·K)^−1^; *β* is the liquid fraction of the PCM (dimensionless). The liquid fraction *β* is determined by the temperature of the grouting material [39,40].

(10) $$\beta =\left\{\begin{array}{ll} 0 & T<T_{f}\\ \frac{T-T_{f}}{T_{m}-T_{f}} & T_{f}<T<T_{m}\\ 1 & T>T_{m} \end{array}\right.,$$

In addition to mass and energy conservation, the momentum conservation must account for the flow resistance caused by the reduced porosity in the mushy zone (partially solidified region of the PCM). This resistance is introduced as a source term in the momentum equation, as expressed in Equation (11) [39,40].

(11) $$S=\frac{{\left(1-\beta \right)}^{2}}{\left(\beta^{3}+\varepsilon \right)}A_{mush}\left(\vec{v}-{\vec{v}}_{p}\right),$$

where *ε* is a small value (less than 0.0001) to avoid division by zero; *A_mush_* is the mushy zone constant; ${\vec{v}}_{p}$ is the velocity of the solid phase in the mushy zone (i.e., the solidification rate), m·s^−1^.

For the turbulence model (Realizable k-ε model) adopted in this study, the momentum source term is modified to adapt to the mushy zone’s porosity change. The corresponding expression is given in Equation (12) [39,40].

(12) $$S=\frac{{\left(1-\beta \right)}^{2}}{\left(\beta^{3}+\varepsilon \right)}A_{mush}\phi ,$$

where *ϕ* is the unknown variable to be solved.

The above governing equations (Equations (7)–(12)) form the complete mathematical model of the PCM-integrated grouting zone.

#### 3.7.4. Boundary Conditions and Parameter Settings

A constant flow rate and fixed inlet water temperature are prescribed at the U-tube inlet. For the summer operating condition, the inlet temperature is set to 35 °C and the flow velocity to 0.6 m/s, while an OUTFLOW boundary condition is adopted at the tube outlet. The initial temperature is uniformly defined as 18 °C for the circulating fluid, U-tube wall, backfill layer, and surrounding soil. Both the outer boundary of the soil domain and the ground surface are treated as adiabatic conditions. Two backfill configurations are adopted in the numerical simulation for comparative analysis: pure OSS and 10% CPCM-doped CPCM/OSS. The thermophysical parameters of the PE pipe, CPCM/OSS composite, and native soil are summarized in Table 8.

#### 3.7.5. Meshing

**Figure 16:**
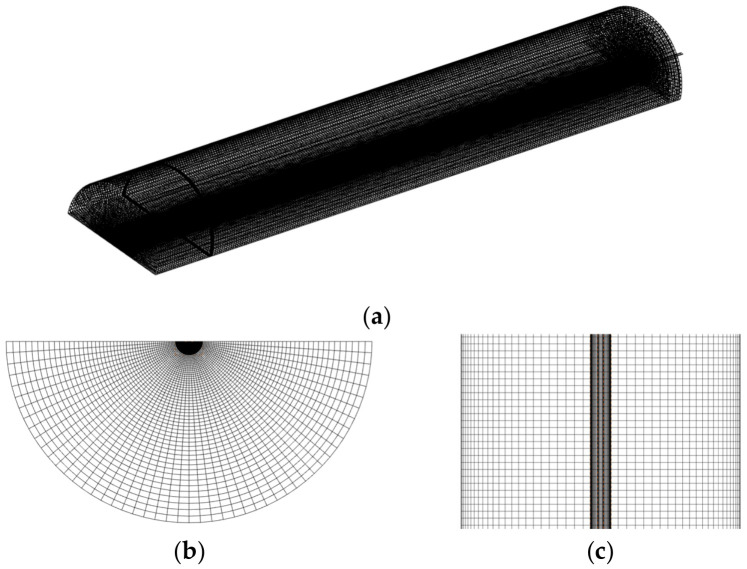
Mesh of vertical single U-Tube Ground: (**a**) Overall grid view; (**b**) Horizontal view; (**c**) Vertical view.

Given the slender structural feature of the model and the insignificant vertical variation of flow characteristics inside the U-tube, a relatively coarse grid spacing is adopted in the vertical direction. In contrast, refined meshing is applied at the bottom U-tube bend, where the fluid flow direction changes sharply. To ensure a smooth mesh transition, the vertical grid is gradually refined toward the bottom bend. On the horizontal cross-section of each component, boundary-layer grids are arranged near the U-tube inner wall. This arrangement enables accurate simulation of convective heat transfer between circulating water and the tube wall. Structured grids are generated for the U-tube wall, whereas dense unstructured grids are adopted for the backfill layer. The soil layer has a much larger radial dimension than the U-tube. Meanwhile, its heat flux and temperature gradient gradually decline along the radial outward direction. Accordingly, the soil domain is discretized with sparse structured grids, and the radial node spacing increases progressively outward. Owing to the axisymmetric distribution of the overall temperature field, only half of the physical model is established for simulation. The detailed mesh configuration is presented in Figure 16.

## 4. Conclusions

This work systematically developed a series of CPCM/OSS backfill materials using CA-MA/EG at mass fractions of 5%, 10%, 15%, and 20%. Their thermophysical properties, phase change behavior, and heat transfer enhancement mechanisms for GHEs in GSHP systems were comprehensively investigated.

(1) The results demonstrate that moisture content and CPCM dosage exert significant positive effects on the thermal transport and storage performance of the backfill. Thermal conductivity increases steadily with rising water content and CPCM loading. Specific heat capacity and thermal storage coefficient are also notably enhanced with increasing CPCM content. DSC results show that detectable phase-change characteristics can be observed at 10 wt% CPCM dosage, and its latent heat just reaches the critical threshold for building thermal storage materials. Based on overall performance, the sample with 10 wt% CPCM is identified as the optimal formulation via a comprehensive trade-off of thermophysical performance, economic cost, mechanical stability, and construction workability.

(2) Laboratory heat transfer experiments show that the CPCM/OSS backfill generates a stronger and more uniform thermal diffusion field around the GHEs than pure OSS. Under identical test conditions, the average soil temperature of the composite backfill group is approximately 2.5–2.8 °C higher than that of pure sand, indicating stronger heat conduction and delayed thermal saturation. Three-dimensional numerical simulations under intermittent summer operation further reveal that the composite backfill reduces vertical temperature stratification, expands the radial heat influence range, and alleviates local thermal accumulation. After three days of cyclic operation, the heat exchange capacity per unit length of the GHEs is improved by 8.8% compared with the OSS backfill.

In summary, the optimized CS-10 composite exhibits prominent high thermal conductivity and good engineering applicability. Its latent heat reaches the practical application threshold and delivers effective auxiliary temperature-buffering performance, while the overall heat transfer enhancement is predominantly governed by thermal conductivity improvement. It serves as a high-performance candidate for GHE backfill applications. This study not only provides a feasible and high-performance backfill material solution for improving the thermal efficiency of GSHP systems but also supplements the thermophysical property data and heat transfer mechanism of binary fatty acid eutectic CPCM/OSS by comparison with conventional single fatty acid and paraffin-based composite phase change materials.

## Figures and Tables

**Table 1:** Specific heat capacity of CPCM/OSS backfill material.

Sample	Specific Heat Capacity/J·(g·K)^−1^	Sample	Specific Heat Capacity/J·(g·K)^−1^
CS-0	1.0251	CS-15	1.5241
CS-5	1.0937	CS-20	1.6964
CS-10	1.1307		

**Table 2:** Heat storage coefficient of CPCM/OSS backfill material.

Sample	Thermal Storage Coefficient/W·(m^2^·K) ^−1^	Sample	Thermal Storage Coefficient/W·(m^2^·K) ^−1^
CS-0	11.3713	CS-15	15.9775
CS-5	11.7114	CS-20	18.6572
CS-10	13.3709		

**Table 3:** Thermal properties of CPCM/OSS backfill material.

Sample	*W* (CPCM) %	Melting		Freezing	
		*T_m_*/°C	*H_m_*/J·g^−1^	*T_f_*/°C	*H_f_*/J·g^−1^
CS-10	10	10.4	5.18	15.9	3.31
CS-15	15	13.5	13.29	17.8	12.15
CS-20	20	15.6	18.99	17.9	14.97

**Table 4:** Quantitative temperature comparison at key measurement points at 15,000 s.

Measuring Point	MP1	MP2	MP3	MP4
Temperature at 15,000 s (°C)—CPCM/OSS	48.2	35.8	28.3	21.5
Temperature at 15,000 s (°C)—OSS	46.5	30.2	22.1	14.3
Temperature Increase (°C)	1.7	5.6	6.2	7.2
Increase Ratio (%)	3.66	18.54	28.05	50.35

**Table 5:** Performance comparison of the proposed CPCM/OSS backfill with reported single fatty acid/paraffin-based PCM backfills for GSHP.

Reference	Material	Phase Change Temperature/°C	Thermal Conductivity	Key Features
[15]	Paraffin RT27 grout	~27	Low	Small thermal influence radius
[16]	Single fatty acid grout	20.4	Medium	Improved system stability
[17]	Microencapsulated PCM soil	–	Decreases with PCM	Higher heat capacity
[18]	Single fatty acid grout	–	Medium	High efficiency in dry soil
[22]	Graphite-enhanced paraffin	29	High	Higher COP improvement
[23]	Shape-stabilized PCM backfill	19.9	Low	Reduces temperature fluctuation
[24]	CA–LA/EG grout	22.5	High	Heat exchange +11.6%
This work	CA–MA/EG + OSS	19.7	1.1086 (High)	Best temperature matching; high thermal conductivity

**Table 6:** Physical and thermal properties of experimental raw materials. Note: Data sources are classified as experimental tests in this study, national standard [33], manufacturer product manuals, and the cited literature [37,38].

Material	Property Index	Value	Data Source
Original sand soil	Original sand soil size range	0.075–2 mm	This study, [33]
	Void ratio	0.23–0.25	
	Dry density	1.5–2.0 g·cm^−3^	
	Natural moisture content	5–15%	
	Thermal conductivity	0.8–1.8 W·(m·K)^−1^	
	Volumetric heat capacity	3.0–3.6 MJ·(m^3^·K)^−1^	
Capric acid	Melting temperature (*T*_m_)	31.4 °C	Manufacturer manual, [37,38]
	Melting enthalpy (*H*_m_)	169.4 J·g^−1^	
	Freezing temperature (*T*_f_)	31.3 °C	
	Freezing enthalpy (*H*_f_)	170.3 J·g^−1^	
Myristic acid	Melting temperature (*T*_m_)	52.7 °C	Manufacturer manual, [37,38]
	Melting enthalpy (*H*_m_)	188.6 J·g^−1^	
	Freezing temperature (*T*_f_)	51.6 °C	
	Freezing enthalpy (*H*_f_)	193.1 J·g^−1^	
Expandable graphite	Mesh number	350	Manufacturer manual
	Expansion rate	100 mL·g^−1^	
	Carbon content	99%	
	Density	1.1 g·cm^−3^	

**Table 7:** Geometric parameters of a U-tube ground-heat exchanger.

Name	Dimension/m	Name	Dimension/m
U-tube inner diameter	0.026	Shaft diameter	0.2
U-tube outer diameter	0.032	Soil radius	2.5
Distance between tubes	0.064	Burial depth	50

**Table 8:** Physical parameters of backfill material.

Parameter Type	OSS	CPCM/OSS	PE Pipe
Thermal Conductivity/W·(m·°C)^−1^	0.8197	1.1086	0.4000
Density/g·cm^−3^	2.063	1.961	0.930
Specific Heat Capacity/J·(g·K)^−1^	1.025	1.131	1.000

## Data Availability

The original contributions presented in this study are included in the article. Further inquiries can be directed to the corresponding authors.
